# A Scaffolded Data-Driven Learning approach to EFL synonym differentiation: an extended unit of meaning perspective

**DOI:** 10.3389/fpsyg.2026.1928278

**Published:** 2026-09-04

**Authors:** Tianfei Ji, Yayun Xi, Xiucai Fang

**Affiliations:** 1 School of Foreign Languages, Huainan Normal University, Huainan, China; 2 School of General Education, Hefei Technology College, Hefei, China

**Keywords:** collocation, concordancing, Data-driven learning, extended unit of meaning, phraseological awareness, synonym differentiation

## Abstract

This study investigates the effectiveness of an Extended Unit of Meaning (EUM)-informed scaffolded Data-Driven Learning (DDL) approach for helping EFL learners differentiate near-synonymous verbs. To support learners’ phraseological induction during concordance observation, the study proposes the Observe–Mark–Write–Induce (OMWI) framework as a scaffolded instructional model. A total of 60 first-year English-major students at a Chinese university participated in the study, with one group receiving EUM-informed DDL-based instruction and the other receiving traditional vocabulary instruction. Pre- and post-tests provided the primary evidence of learning gains, while a post-treatment questionnaire supplied complementary evidence concerning learners’ perceived awareness of collocation, colligation, and semantic preference. The results showed that learners receiving EUM-informed DDL-based instruction achieved significantly greater gains in synonym differentiation than those receiving traditional instruction. The questionnaire responses further suggest that learners perceived the OMWI-supported concordancing activities as helpful for noticing recurrent usage patterns and understanding contextual differences between near-synonymous verbs. Although learners reported certain challenges in interpreting concordance evidence, they generally expressed positive attitudes toward DDL-based synonym learning. The study contributes to corpus-informed vocabulary instruction by showing how scaffolded concordance observation can direct learners’ attention to phraseological patterns and support synonym differentiation in EFL classrooms.

## 1 Introduction

Vocabulary learning involves not only understanding the core meanings of words, but also distinguishing how semantically related words are used in authentic contexts. Near-synonymous words represent a persistent challenge for EFL learners because semantic similarity does not necessarily imply interchangeability. Learners may understand the core meanings of synonymous items while still making inappropriate lexical choices in authentic contexts. Such difficulties frequently arise not from misunderstanding dictionary definitions, but from insufficient awareness of collocation, grammatical patterning, semantic preference, and contextual constraints. In this sense, synonym differentiation concerns deeper dimensions of lexical knowledge rather than vocabulary breadth alone.

Corpus Linguistics, Data-Driven Learning (DDL), and the Extended Unit of Meaning (EUM) provide complementary perspectives for addressing such problems (Johns, 1991a; Sinclair, 2004). DDL encourages learners to explore authentic language data through concordancing and identify recurring lexical and grammatical patterns through direct observation. EUM further explains why such recurrent patterns are central to lexical meaning: words do not function as isolated units, but acquire meaning through repeated collocational, grammatical, and semantic associations. In this sense, integrating DDL and EUM is valuable for synonym differentiation. DDL provides the pedagogical means for observing corpus evidence, while EUM provides the theoretical lens for interpreting the phraseological patterns revealed by that evidence. Previous studies have demonstrated the effectiveness of DDL in vocabulary learning, collocation acquisition, and phraseological awareness (Boulton and Cobb, 2017; Lee et al., 2019; Liu and Gablasova, 2025; Li, 2025). However, fewer studies have examined how EUM-informed DDL can be implemented in classroom-based synonym instruction to help learners identify collocational, colligational, and semantic preference patterns simultaneously. Another important issue concerns the cognitive demands of concordance observation. Although DDL is often associated with learner autonomy and discovery learning, interpreting concordance lines may be challenging for novice corpus users. This challenge highlights the importance of pedagogical scaffolding in classroom concordancing activities (Corino and Onesti, 2019; O’Keeffe, 2021; Tosun and Sofu, 2023).

Against this background, the present study investigates EFL learners’ synonym differentiation through an EUM-informed DDL approach. It proposes the Observe–Mark–Write–Induce (OMWI) framework as a scaffolded concordancing model designed to guide learners in observing corpus evidence, marking salient patterns, recording findings, and inducing phraseological generalizations. In doing so, the study makes three contributions. First, it shows how an EUM perspective can inform the selection and interpretation of concordance evidence in classroom-based synonym instruction. Second, it operationalizes this perspective through a pedagogically scaffolded procedure for concordance observation. Relative to ICG, OHE, and OH-IC, OMWI makes two intermediate scaffolds explicit: Mark externalizes noticing through visual annotation, while Write organizes the noticed evidence into EUM-based categories before induction. Third, the study provides empirical evidence on whether this approach can promote learners’ synonym differentiation and their perceived awareness of patterns of collocation, colligation, and semantic preference associated with near-synonymous verbs.

## 2 Literature review

### 2.1 Corpus-based approaches to synonym differentiation

Etymologically, the word corpus (plural corpora) derives from Latin and originally refers to “body” or “a collection of things”. According to Sinclair (1991), a corpus is generally understood as a collection of naturally occurring language data compiled for linguistic investigation. Similarly, Crawford and Csomay (2015) define a corpus as “a representative collection of language that can be used to make statements about language use” (p. 6). Corpus Linguistics focuses on the scientific analysis and research of languages using corpus data. It treats language as a large-scale, data-driven phenomenon that can be objectively examined and quantified. By allowing research based on authentic samples of language use, Corpus Linguistics aims to uncover underlying patterns and trends in language usage more precisely.

The study of near-synonymous expressions has benefited considerably from corpus-based approaches. Previous studies have examined synonym distinction through analyses of collocational, grammatical, semantic, and contextual usage patterns (Xiao and McEnery, 2006; Thongpan, 2022; Alanazi, 2022; Tang, 2025). For example, Xiao and McEnery (2006) explored collocation, semantic prosody, and near synonymy from a cross-linguistic perspective, demonstrating that near-synonymous expressions may differ substantially in their collocational and semantic tendencies despite apparent semantic similarity. Similarly, Thongpan (2022) showed that the synonymous adjectives *far*, *distant*, and *remote* differ in collocation, semantic preference, degree of formality, and contextual usage. Alanazi (2022) further examined the near-synonymous verbs *affect* and *impact* and found systematic differences in their collocational, colligational, and semantic preference patterns. Taken together, these studies suggest that corpus-informed approaches can reveal subtle phraseological distinctions that are often difficult to capture through dictionary explanations alone.

### 2.2 Data-Driven Learning (DDL) and synonym differentiation

Within the realm of foreign language education and learning, Johns (1991a) introduced the term “Data-Driven Learning” (DDL), arguing that “the language-learner is also, essentially, a research worker whose learning needs to be driven by access to linguistic data” (p. 2). Johns (1991a) further divided the DDL process into three stages: Identify-Classify-Generalize (ICG). Johns (1991b) further showed that concordance output could be transformed from printouts into classroom handouts for grammar and vocabulary teaching, indicating that DDL can be implemented in teacher-mediated classroom settings rather than only through open-ended learner corpus searches. In Lewis’s (1993) DDL process, three phases of language learning were identified: Observe, Hypothesize, Experiment (OHE). OHE is generally regarded as an inductive approach that relies on learners’ observation, reflection, and hypothesis formation. According to Golebiewska and Jones (2018), OHE lessons tend to focus on receptive knowledge of new language rather than its productive use, emphasizing repeated exposure over an extended period of time. On the basis of OHE, Xu (2009) further summarized the procedures for a corpus-based approach to vocabulary instruction as OH, IC (Observe-Hypothesize-Induce from Corpus). Unlike ICG, OHE, and “OH, IC” sequences, OMWI makes two intermediate scaffolds between observation and induction explicit. Mark externalizes noticing through visual annotation, while Write organizes the identified evidence according to the EUM categories of collocation, colligation, and semantic preference before learners formulate a generalization (Table 1).

**Table 1:** Representative DDL procedures and the OMWI extension.

Procedures of DDL	Proposer
Identify-Classify-Generalize (ICG)	Johns (1991a)
Observe, Hypothesize, Experiment (OHE)	Lewis (1993)
Observe-Hypothesize-Induce from Corpus (OH, IC)	Xu (2009)
Observe–Mark–Write–Induce (OMWI)	Present study

Since its introduction, DDL has developed into a substantial area of corpus-informed pedagogy, with 30-year review evidence and meta-analytic evidence generally supporting its effectiveness across L2 learning contexts (Boulton and Vyatkina, 2021; Boulton and Cobb, 2017; Lee et al., 2019; Liu and Ma, 2025). Recent work has further broadened this evidence base. Pérez-Paredes and Boulton (2025) emphasize language awareness, noticing, and critical engagement with corpus evidence, while Flowerdew (2025) traces DDL from the COBUILD tradition to emerging applications involving large language models. Focusing on pre-tertiary learners, Huang and Ma’s (2025) systematic review of 45 studies reports broadly positive findings across age groups and types of corpus interaction. Early work showed that concordance-based activities could support vocabulary learning and lexical awareness (Stevens, 1991). Subsequent empirical studies further demonstrated the value of DDL for vocabulary acquisition, retention, collocation learning, and phraseological development. For example, Soruç and Tekin (2017) reported that DDL facilitated vocabulary learning and was generally well received by learners; Lee and Lin (2019) found that both inductive and deductive DDL approaches contributed positively to vocabulary acquisition and retention; Liu and Gablasova (2025) provided longitudinal evidence that DDL can support collocation learning over time; and Li (2025) showed that DDL can enhance collocational accuracy when integrated with indirect corrective feedback.

The evidence is nevertheless not uniformly positive. Shin and Lee (2016), for example, found that corpus-based instruction did not outperform traditional instruction in the acquisition or retention of receptive or productive vocabulary knowledge among beginning-level learners. Lee and Lin (2019) found inductive and deductive DDL to be equally effective for the acquisition and retention of word form, meaning, and use. Taken together, these findings caution against assuming that concordance exposure or a purely inductive format will necessarily produce superior outcomes.

Despite its pedagogical benefits, DDL also presents challenges for learners and teachers. Concordance observation requires learners to interpret KWIC lines, identify recurring patterns, and generalize linguistic tendencies from authentic language data. Such processes may impose considerable cognitive demands, particularly on novice corpus users, and learner factors such as vocabulary proficiency, strategy use, and working memory may further shape the success of corpus-based vocabulary learning (Lee et al., 2020). These demands are especially relevant to synonym differentiation, where learners must compare two semantically related items across multiple contexts rather than learn the meaning of a single word in isolation. Previous research has therefore emphasized the importance of guided corpus use, carefully curated materials, and explicit scaffolding in classroom DDL implementation (Corino and Onesti, 2019; O’Keeffe, 2021). Zare et al. (2022) also reported that learners often relied heavily on teacher guidance during corpus consultation activities, while Tosun and Sofu (2023) found that lower-proficiency learners required substantial instructional support when engaging in concordance observation tasks. Pedagogical design therefore needs to mediate authentic corpus evidence through pre-selected concordance lines, guided annotation, and structured recording tasks.

One potential means of supporting concordance observation is annotation. Through marking recurrent lexical and grammatical patterns, learners may focus attention more effectively on relevant language features and compare corpus evidence more systematically. Johns (1988) observed that “multicoloured fibre-tip pens for marking up contexts are an excellent auxiliary tool” (p. 24), linking coloured marking to learners’ practice in identifying and classifying concordance patterns. This suggests that coloured annotation may serve as a simple but useful form of visual scaffolding during concordance analysis. Such marking activities may also facilitate noticing, which has been widely recognized as an important condition for second language learning (Schmidt, 1990).

Existing research has demonstrated that DDL can effectively promote vocabulary learning and phraseological awareness (Boulton and Cobb, 2017; Lee et al., 2019; Soruç and Tekin, 2017; Liu and Gablasova, 2025; Li, 2025). Nevertheless, several limitations remain. First, relatively few studies have integrated DDL with Sinclair’s (2004) Extended Unit of Meaning (EUM) framework when investigating vocabulary learning. Second, although synonym differentiation has frequently been examined through corpus-based approaches (Xiao and McEnery, 2006; Thongpan, 2022; Alanazi, 2022; Tang, 2025), fewer studies have simultaneously investigated collocation, colligation, and semantic preference as interconnected dimensions of lexical behavior. Third, while previous studies have highlighted the cognitive demands associated with concordance observation (Corino and Onesti, 2019; O’Keeffe, 2021; Zare et al., 2022; Tosun and Sofu, 2023), limited attention has been paid to the design of scaffolded classroom procedures that support learners’ phraseological induction during corpus consultation.

To address these gaps, the present study investigates DDL-based synonym learning from an Extended Unit of Meaning perspective. Drawing on the phraseological dimensions of collocation, colligation, and semantic preference, the study proposes the Observe–Mark–Write–Induce (OMWI) framework as a scaffolded classroom concordancing model for supporting learners’ observation and induction of recurrent lexical patterns while reducing the cognitive demands associated with classroom DDL activities. Building on earlier DDL procedures such as ICG, OHE, and the “OH, IC” sequence, OMWI retains their observation–induction logic but makes two intermediate scaffolding operations explicit. Mark externalizes noticing through visual annotation, while Write stabilizes and coordinates observations in an EUM-organized record before induction. These added stages are important because EUM-informed synonym learning requires learners to coordinate several kinds of evidence: the words that recurrently occur with a node, the grammatical environments in which the node appears, and the semantic domains toward which the node tends to be attracted. These stages guide learners to identify collocational, colligational, and semantic preference patterns in concordance data, an emphasis consistent with prior DDL research showing that structured guidance can mediate learners’ engagement with corpus evidence (Smart, 2014).

The study addresses the following research questions:

*RQ1*. How does EUM-informed scaffolded concordancing through the OMWI framework affect EFL learners’ differentiation of near-synonymous verbs compared with traditional vocabulary instruction?

*RQ2*. How do learners perceive the role of the OMWI framework in supporting their awareness of collocation, colligation, and semantic preference during concordance-based synonym learning?

## 3 Method

### 3.1 Participants

Before the instructional treatment, an initial pretest was administered to 141 first-year English-major students at a university in China. Based on the distribution of their pretest scores, all 141 students were first stratified into high-, intermediate-, and low-performing bands. From this stratified pool, 60 students were selected to ensure representation across the three performance bands. To enhance baseline comparability, the selected students were then randomly allocated within each band to either the Control Group or the Experimental Group, yielding 30 participants in each group. The Control Group comprised 3 male and 27 female students (mean age = 19.47, SD = 0.76), while the Experimental Group comprised 1 male and 29 female students (mean age = 19.83, SD = 1.07). All participants were native speakers of Chinese and learners of English as a foreign language, and none had received formal instruction in corpus linguistics or DDL. An independent-samples *t*-test confirmed that the two groups did not differ significantly in their pretest scores.

Ethical approval was obtained from the Ethics Committee of the corresponding author’s institution. All participants provided written informed consent prior to participation in the study. Participation was voluntary, and participants were informed of their right to withdraw at any time without penalty. The study was conducted in accordance with the ethical standards of the institutional and national research committees and with the 1964 Declaration of Helsinki and its later amendments.

### 3.2 Research design

The study adopted a quasi-experimental between-group pretest-posttest design to examine whether EUM-informed DDL-based scaffolded concordancing would facilitate EFL learners’ differentiation of near-synonymous verbs more effectively than traditional vocabulary instruction. The design compared two instructional conditions: an experimental condition using EUM-informed concordance observation through the OMWI framework and a control condition using teacher explanation, dictionary consultation, and practice activities.

### 3.3 Materials and Corpus resources

The target words of the present study consisted of five pairs of synonymous English verbs: *testify* and *validate*, *issue* and *release*, *cure* and *heal*, *recover* and *revive*, and *wish* and *hope*. Only the verbal usages of the target words were examined in order to ensure that the instructional activities focused specifically on synonym differentiation within authentic contexts of use. Instruction in the Control Group primarily relied on the *Oxford Advanced Learner’s English-Chinese Dictionary* and the *Collins COBUILD Advanced Learner’s English-Chinese Dictionary*, which provided definitions, translations, and example sentences for the target synonym pairs.

**Figure 1:**
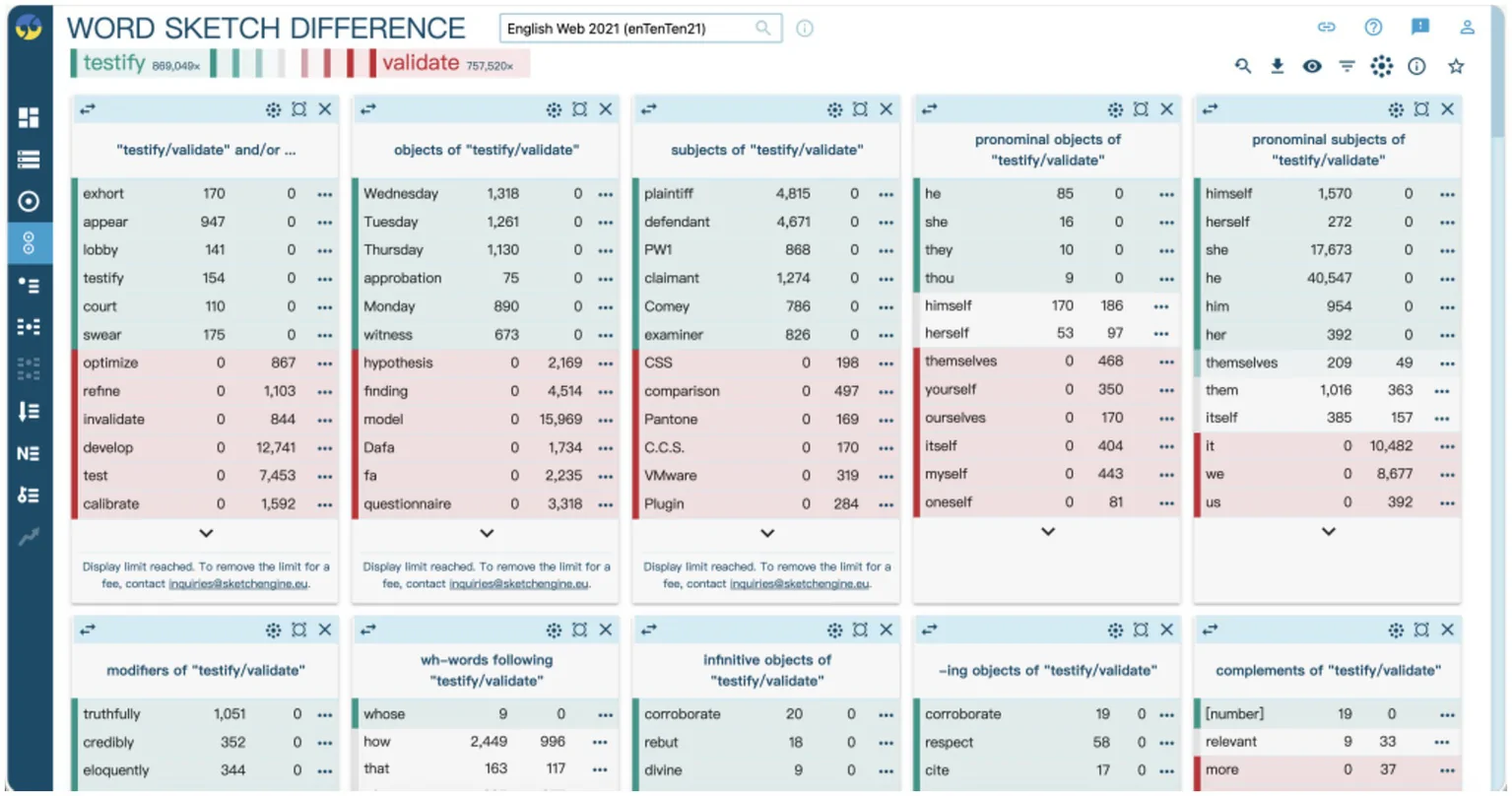
Word Sketch Difference between “testify” and “validate” in Sketch Engine. Kilgarriff et al. (2014). Source: Sketch Engine, https://www.sketchengine.eu/.

**Figure 2:**
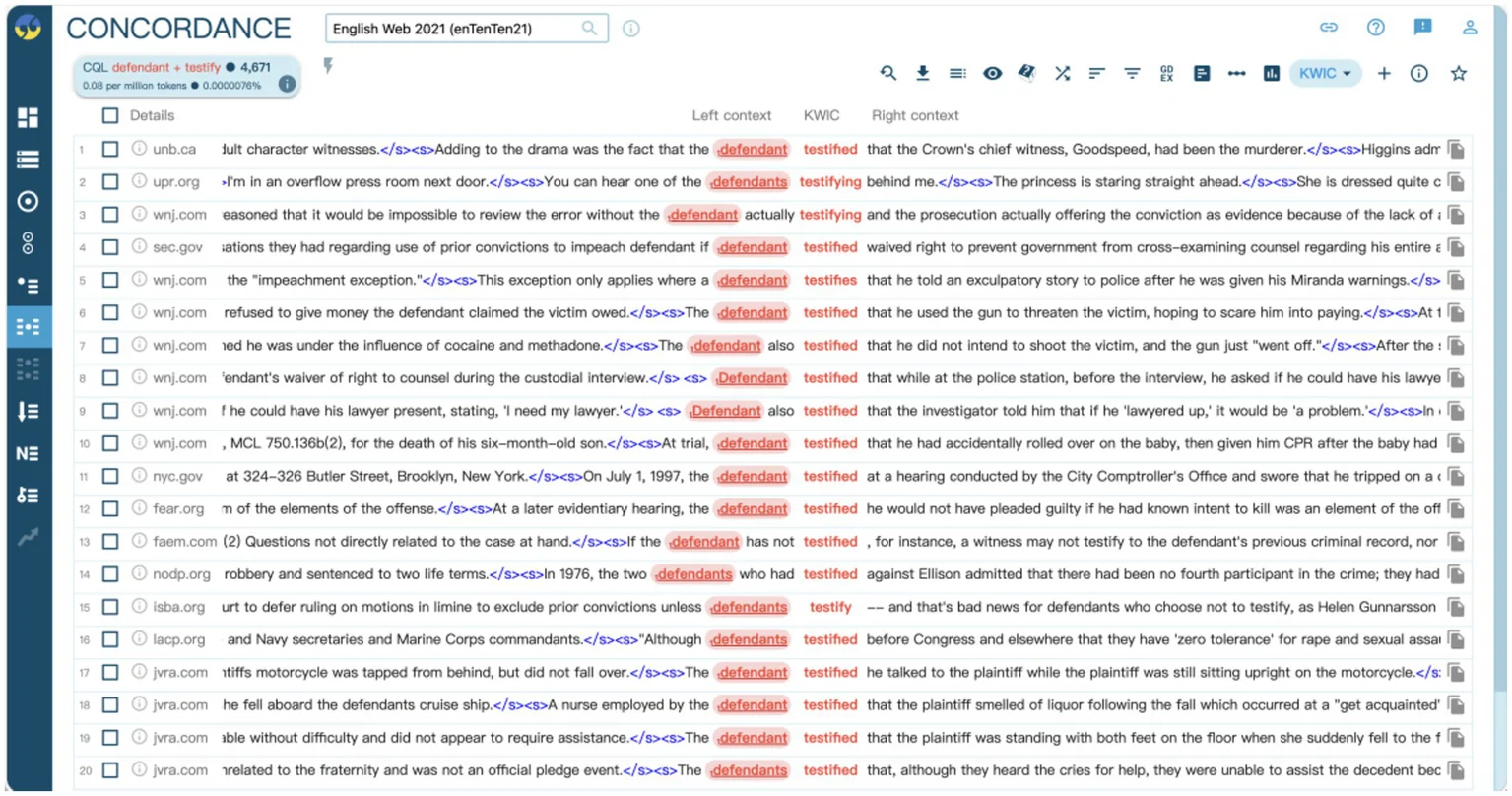
KWIC concordance lines for “defendant + testify” in Sketch Engine. Kilgarriff et al. (2014). Source: Sketch Engine, https://www.sketchengine.eu/.

The instructional materials for the Experimental Group were prepared from the enTenTen21 corpus, a large web-based corpus of approximately 52 billion words available through Sketch Engine (Kilgarriff et al., 2014). Before the treatment, the researcher used Sketch Engine tools, including Concordance, Word Sketch, and Word Sketch Difference, to retrieve and compare recurrent usage patterns associated with the target synonym pairs, with particular attention to collocation, colligation, and semantic preference. Because all target items were verbs, the selection of concordance materials focused mainly on patterns involving subjects, objects, pronouns, and verb-preposition combinations, which were considered especially relevant to distinguishing the target pairs in use. Concordance lines exhibiting clear and pedagogically interpretable patterns of collocation, colligation, and semantic preference were then manually selected and organized for classroom use. The classroom tasks centered on interpreting these prepared concordance materials rather than conducting open-ended corpus searches in Sketch Engine. Figure 1 presents an example of Word Sketch Difference analysis for “testify” and “validate.” The contrasting collocational profiles generated by Sketch Engine helped identify usage patterns associated with each verb and informed the selection of target synonym pairs. Figure 2 illustrates KWIC concordance lines for “defendant + testify”, showing how recurrent lexical and grammatical patterns were extracted from authentic corpus data and prepared for classroom observation. The instructional materials for the Experimental Group also included the bilingual DDL handout, printed or displayed concordance lines, structured summary tables for recording collocational and colligational observations, and multi-coloured pens for marking salient patterns during concordance activities. The summary tables required learners to record recurring collocates and grammatical environments before formulating semantic preferences, thereby linking the classroom task format directly to the EUM dimensions targeted in the study.

### 3.4 Instruments

The study employed a pretest and a posttest to measure learners’ ability to distinguish the target synonym pairs in contextualized sentence environments. Both tests adopted the same format and consisted of multiple-choice items constructed from authentic corpus examples retrieved from enTenTen21 with the assistance of the *Good Dictionary Examples (GDEX)* function in Sketch Engine.

A post-treatment questionnaire was designed by the authors and was additionally administered to participants in the Experimental Group in order to investigate learners’ perceptions of DDL-based synonym learning. The questionnaire contained 20 five-point Likert-scale items organized into five content domains: comprehension of phraseological and procedural terms (S1–S4), perceived ability to observe concordance evidence (S5–S8), attitudes toward the DDL procedures (S9–S12), perceived effectiveness (S13–S17), and willingness to use DDL in the future (S18–S20). Representative items included S5, “I could autonomously find collocations by observing concordances”; S6, “I could autonomously find colligations by observing concordances”; and S16, “I believe that DDL is effective for distinguishing synonyms”.

The questionnaire responses were analyzed descriptively to examine learners’ self-reported perceptions rather than to measure gains in collocation, colligation, or semantic preference directly, so no validity or reliability analysis was conducted.

### 3.5 Procedure

Before the formal experiment, a pilot study was conducted to examine the feasibility of the instructional procedures and identify potential difficulties associated with classroom concordancing. Five English-major students participated in a small-scale DDL class involving concordance observation and synonym differentiation tasks. The pilot study revealed that the participants initially experienced considerable difficulty interpreting concordance lines independently. Most participants attempted to read concordance lines in the same manner as ordinary sentences rather than treating them as contextual evidence for phraseological observation, and they also reported confusion when attempting to identify more abstract linguistic features such as colligation and semantic preference. Based on these observations, the procedure was revised so that the first synonym pair was completed through teacher-guided demonstration before learners attempted the remaining pairs more independently; this revision was intended to make the OMWI steps clearer and reduce the cognitive burden associated with DDL tasks.

The research procedure lasted approximately 4 weeks. The first 3 weeks were devoted to the selection and preparation of instructional materials. During this stage, a large number of near-synonymous verbs were examined through Sketch Engine, and target pairs were selected based on corpus-derived patterns relevant to EUM differentiation. For each target verb, 40 carefully selected concordance lines were prepared for classroom observation.

The formal procedure consisted of four stages. First, all candidates completed the pretest, and two comparable groups were formed on the basis of their initial performance. Second, the instructional treatment was conducted separately for the Control Group and the Experimental Group. Third, both groups completed the posttest after the treatment. Finally, participants in the Experimental Group completed the post-treatment questionnaire, which elicited their views on the OMWI procedures and their perceived understanding of EUM-related patterns.

In the Control Group, vocabulary instruction followed a relatively traditional Presentation-Practice-Production sequence commonly used in vocabulary teaching. The teacher first introduced each target synonym pair through dictionary definitions, Chinese translations, and example sentences, and learners then completed gap-filling exercises and sentence-making activities designed to reinforce distinctions between the target words. The entire instructional procedure, including vocabulary instruction and the posttest, lasted approximately 20 min.

In the Experimental Group, the treatment began with corpus training supported by a PowerPoint presentation, a bilingual instructional handout, and an instructor-led demonstration of the Sketch Engine interface. Because most learners had little prior experience with corpora or concordancing, the instructor introduced several foundational concepts, including corpus, concordance line, KWIC, node word, collocation, colligation, and semantic preference. The handout provided concise bilingual explanations of these terms and guided learners to treat concordance lines as evidence for observing recurrent patterns rather than as ordinary sentences to be translated. The initial corpus training lasted approximately 20 min.

The OMWI framework consisted of four interrelated stages designed to scaffold learners’ phraseological induction during classroom concordancing. First, in the Observe stage, learners examined concordance lines for each synonym pair and attended to recurring lexical and grammatical patterns surrounding the node word, especially nouns, pronouns, and prepositions occurring before or after the verb. Second, in the Mark stage, learners used coloured annotation to circle, underline, or otherwise highlight salient patterns in the prepared concordance lines, making recurrent collocational and colligational features more visually noticeable. Echoing Johns’ experience, multi-coloured pens were distributed as auxiliary instruments to support this process. Third, in the Write stage, learners recorded recurrent collocations and colligational patterns in structured summary tables and used these records to note emerging semantic preferences associated with each target word. Finally, in the Induce stage, learners drew on the evidence they had observed, marked, and written down to formulate broader semantic preferences and usage distinctions between the target synonym pairs.

After introducing the OMWI procedures to the learners, the instructor demonstrated the complete process using the synonym pair *testify* and *validate*. The demonstration showed learners how to locate a node word, inspect KWIC concordance lines, mark relevant lexical and grammatical clues, complete the summary table, and move from observed patterns to EUM-informed generalizations. This teacher-guided demonstration and practice session lasted approximately 20 min. Following this session, learners independently applied the OMWI procedures to the remaining synonym pairs. The entire Experimental Group session, including corpus training, teacher-guided demonstration and practice, autonomous application of the OMWI procedures to the remaining synonym pairs, the posttest, and the post-treatment questionnaire, lasted approximately 90 min.

### 3.6 Data analysis

The quantitative data of the present study consisted primarily of pretest and posttest scores collected from both groups. Statistical analyses were conducted using SPSS 26.0. To examine whether the Experimental Group improved significantly more than the Control Group from pretest to posttest, a 2 × 2 mixed-design ANOVA was conducted, with Group (Experimental vs. Control) as the between-subjects factor and Time (pretest vs. posttest) as the within-subjects factor. The Group × Time interaction served as the primary test of interest because it directly compares the magnitude of pretest–posttest change between the two groups. Because Time had only two levels, sphericity was automatically satisfied and no correction (e.g., Greenhouse–Geisser) was required. The assumptions of the mixed ANOVA were checked prior to the analysis: Shapiro–Wilk tests on the pretest–posttest gain scores indicated no significant departure from normality in either group (Control: *w* = 0.964, *p* = 0.380; Experimental: *w* = 0.967, *p* = 0.470), and Levene’s tests indicated homogeneity of variances for both the pretest (*f* = 0.99, *p* = 0.324) and the posttest (*f* = 1.74, *p* = 0.193) scores. As a preliminary check of baseline comparability, an independent-samples *t*-test was conducted on the pretest scores. Effect sizes were reported as partial eta-squared ( $\eta_{p}^{2}$ ) for the ANOVA effects and as Cohen’s d for the between-group gain comparison, computed by dividing the mean difference in gains by the pooled standard deviation; 95% confidence intervals were reported for the mean gain difference.

The questionnaire data were analyzed descriptively and used as explanatory evidence for the two research questions. For RQ1, the pretest-posttest results provided the primary evidence of learning gains, while questionnaire items on perceived effectiveness and learning experience (especially S13–S17 and S9–S12) helped interpret why the Experimental Group may have benefited from scaffolded concordancing. For RQ2, questionnaire items on learners’ perceived understanding of collocation, colligation, semantic preference, and the OMWI procedures (S1–S8) provided the primary evidence, while the contextualized test performance was used as complementary outcome evidence.

## 4 Results

### 4.1 Effects of EUM-informed DDL-based scaffolded concordancing on synonym differentiation

Before the instructional treatment, the two groups were comparable in their pretest performance. The mean pretest score was 52.47 (SD = 7.44) for the Control Group and 52.80 (SD = 8.58) for the Experimental Group, and an independent-samples *t*-test confirmed that the difference was not significant, *t* (58) = −0.16, *p* = 0.873 (Levene’s test: *F* = 0.99, *p* = 0.324).

To examine the effects of the two instructional conditions on synonym differentiation, a 2 × 2 mixed-design ANOVA was conducted on the pretest and posttest scores, with Group as the between-subjects factor and Time as the within-subjects factor. Table 2 presents the descriptive statistics, and Table 3 summarizes the ANOVA results. The main effect of Time was significant, *F* (1, 58) = 104.94, *p* < 0.001, partial $\eta_{p}^{2}$ = 0.64, indicating that learners in both groups improved substantially from pretest to posttest. The main effect of Group was not significant, *F* (1, 58) = 3.47, *p* = 0.068, partial $\eta_{p}^{2}$ = 0.06. Critically, the Group × Time interaction was significant, *F* (1, 58) = 9.75, *p* = 0.003, partial $\eta_{p}^{2}$ = 0.14, indicating that the Experimental Group’s pretest–posttest gain (*M* = 15.27, SD = 9.52) was significantly larger than the Control Group’s gain (*M* = 8.13, SD = 8.12). The mean gain difference of 7.13 points was accompanied by a 95% confidence interval of [2.56, 11.71] and corresponded to a medium-to-large effect (Cohen’s *d* = 0.81 for the gain-score comparison).

**Table 2:** Descriptive statistics for pretest and posttest scores by group.

Group	*n*	Pretest M (SD)	Posttest M (SD)
Control group	30	52.47 (7.44)	60.60 (9.05)
Experimental group	30	52.80 (8.58)	68.07 (11.42)

**Table 3:** Two-way mixed ANOVA results for pretest and posttest scores.

Source	df	*F*	*p*	Partial $\eta_{p}^{2}$
Group	1, 58	3.47	0.068	0.06
Time	1, 58	104.94	< 0.001	0.64
Group × Time	1, 58	9.75	0.003	0.14
Error (between subjects)	58			
Error (within subjects)	58			

Overall, both groups improved from pretest to posttest, but the significant Group × Time interaction indicates that the improvement was substantially greater in the Experimental Group than in the Control Group. Descriptively, the posttest difference between the two groups (Experimental: *M* = 68.07, SD = 11.42; Control: *M* = 60.60, SD = 9.05) corresponded to Cohen’s *d* = 0.72.

### 4.2 Questionnaire evidence for learning gains and EUM-related pattern awareness

Table 4 presents the descriptive statistics for the 28 valid questionnaire responses.

**Table 4:** Questionnaire data.

Dimension	Statement	Mean	SD
Comprehension of related terms	S1. I could comprehend what collocation is.	4.14	0.80
	S2. I could comprehend what colligation is.	3.04	1.04
	S3. I could comprehend what semantic preference is.	3.43	0.88
	S4. I could comprehend the OMWI procedures.	4.21	0.79
Ability to observe concordances	S5. I could autonomously find collocations by observing concordances.	3.71	0.66
	S6. I could autonomously find colligations by observing concordances.	2.86	0.65
	S7. I could autonomously induce semantic preferences by observing concordances.	3.07	0.60
	S8. I could autonomously follow the OMWI procedures.	3.64	0.56
Attitudes toward DDL procedures	S9. I felt time-consuming when learning vocabulary by observing concordances.	2.75	1.00
	S10. I felt anxious when inducing language patterns by observing concordances.	2.71	1.01
	S11. I felt challenging when inducing language patterns by observing concordances.	3.36	0.95
	S12. I felt interesting when inducing language patterns by observing concordances.	3.54	0.92
Attitudes toward the effectiveness of DDL	S13. I believe that finding collocations by observing concordances is effective for vocabulary acquisition.	4.00	0.77
	S14. I believe that finding colligations by observing concordances is effective for vocabulary acquisition.	4.04	0.88
	S15. I believe that inducing semantic preferences by observing concordances is effective for vocabulary acquisition.	3.93	0.86
	S16. I believe that DDL is effective for distinguishing synonyms.	4.36	0.78
	S17. I believe that DDL is more effective in distinguishing synonyms than the traditional teaching approach and learning strategy.	3.96	0.79
Future willingness to engage with DDL	S18. I hope to be exposed to more DDL-based classes in the future.	3.50	0.96
	S19. I would be willing to try using DDL for vocabulary learning in the future.	3.25	0.84
	S20. If I become an English teacher in the future, I would be willing to try using DDL for vocabulary teaching.	2.64	0.68

For “Comprehension of related terms” (S1–S4), mean scores ranged from 3.04 to 4.21. Participants reported relatively strong understanding of collocation (S1, *M* = 4.14) and the OMWI procedure (S4, *M* = 4.21), whereas colligation received the lowest rating in this dimension (S2, *M* = 3.04). This pattern indicates comparatively weaker perceived understanding of colligation.

For “Ability to observe concordances” (S5–S8), participants rated their ability to identify collocations (S5, *M* = 3.71) and follow OMWI independently (S8, *M* = 3.64) more highly than their ability to identify colligations (S6, *M* = 2.86) or induce semantic preferences (S7, *M* = 3.07).

The third dimension, “Attitudes toward DDL procedures” (S9–S12), showed mixed results. The mean scores for S9 (2.75) and S10 (2.71) were below 3.0, suggesting that participants generally did not strongly perceive the DDL procedures as overly time-consuming or anxiety-inducing. Meanwhile, the mean scores for S11 (3.36) and S12 (3.54) indicate that learners generally found the process of observing concordances both challenging and interesting.

Moving on to the fourth dimension “Attitudes toward the effectiveness of DDL” (S13–S17), the mean scores were generally high, indicating that the participants believed that finding collocations, colligations, and inducing semantic preferences by observing concordances are effective for vocabulary acquisition. In particular, the mean score for S16 (4.36) was the highest, indicating that the participants believed that DDL is effective for distinguishing synonyms. The mean score for S17 (3.96) was also relatively high, indicating that the participants perceived DDL as being more effective for synonym differentiation than traditional vocabulary instruction.

Finally, in the fifth dimension “Future Willingness to Engage with DDL” (S18–S20), participants were less positive about future DDL use. For S18, the mean score of 3.50 suggests that the respondents hoped to be exposed to more DDL-based classrooms in the future. However, the mean score of 3.25 for S19 suggests slightly lower willingness to use DDL for their own future vocabulary learning, and the mean score of 2.64 for S20 indicates some reluctance to use DDL for vocabulary teaching if they become English teachers in the future. Notably, S20 was the lowest-rated item in the questionnaire.

## 5 Discussion

### 5.1 EUM-informed DDL and synonym differentiation (RQ1)

With respect to RQ1, the 2 × 2 mixed ANOVA revealed a significant Group × Time interaction, *F* (1, 58) = 9.75, *p* = 0.003, partial $\eta_{p}^{2}$ = 0.14, indicating that the Experimental Group’s pretest–posttest gain (*M* = 15.27) was significantly larger than the Control Group’s gain (*M* = 8.13), with a mean gain difference of 7.13 points (95% CI [2.56, 11.71]; Cohen’s *d* = 0.81). These results accord with meta-analytic evidence that corpus use can support L2 vocabulary learning (Boulton and Cobb, 2017; Lee et al., 2019) and with longitudinal evidence for DDL-supported collocation learning (Liu and Gablasova, 2025). However, they contrast with Shin and Lee’s (2016) finding that corpus-based instruction did not outperform traditional vocabulary teaching.

One possible explanation concerns the degree of instructional mediation: the present treatment combined pre-selected concordance lines, teacher demonstration, visual annotation, and structured written recording. These supports may have helped novice learners manage the cognitive demands of concordance analysis and convert recurring corpus evidence into usable synonym distinctions. Given the quasi-experimental design, however, the findings indicate an instructional advantage in the present setting and should not be generalized as a context-independent causal effect.

The findings also have implications for the relationship between DDL and EUM proposed in the Introduction. From an EUM perspective, synonym differentiation depends on recognizing that lexical meaning is distributed across recurrent phraseological relations rather than located in the node word alone. The Experimental Group’s advantage is consistent with the view that comparing collocation, colligation, and semantic preference can make restrictions on synonym interchangeability more accessible to learners. At the same time, the findings refine the discovery-learning principle of DDL: access to concordance evidence may not by itself lead novice learners to phraseological generalizations. In OMWI, Mark turns salient features into visible evidence, while Write reorganizes that evidence according to EUM dimensions before induction. These operations therefore provide a theoretical bridge between DDL as a process of reasoning from data and EUM as an account of the phraseological organization of lexical meaning.

### 5.2 Learners’ perceived EUM-related pattern awareness (RQ2)

With respect to RQ2, collocation was the most accessible of the three EUM dimensions. Participants reported relatively strong understanding of collocation and greater confidence in identifying collocations than colligations or semantic preferences. This pattern is consistent with the comparatively observable nature of repeated lexical co-occurrence in KWIC lines: recurrent words near the node can be marked directly and compared across examples. In theoretical terms, collocation provides the most immediately visible indication that lexical meaning extends beyond the individual word, making it a plausible entry point into EUM-informed analysis.

Colligation presented a different pattern. Although participants rated the usefulness of identifying colligations positively, their self-reported understanding and ability to identify them were lower. This gap between perceived value and perceived ability suggests that recognizing grammatical patterning requires more than noticing repeated surface forms; learners must abstract across features such as subject, object, complementation, and prepositional environments. The finding therefore supports the role of Write as an intermediate scaffold that groups dispersed grammatical evidence before induction, while also indicating that this EUM dimension may require more explicit teacher mediation.

Semantic preference occupied an intermediate position. Participants generally understood and valued this dimension, but were less confident in inducing it independently than in identifying collocations. Unlike collocation, semantic preference requires learners to move from individual lexical items to broader semantic domains shared by recurrent collocates. This additional level of categorization helps explain why semantic preference is theoretically central to EUM yet cognitively less transparent in concordance work. The Induce stage is intended to support this movement from local examples to a generalized semantic association, although the questionnaire evidence reflects perceived awareness rather than demonstrated mastery.

The dimension-specific pattern clarifies the theoretical role of OMWI. The sequence does not treat phraseological awareness as a single undifferentiated outcome; it supports a progression from visible lexical recurrence, through grammatical abstraction, to semantic generalization. Mark is most directly aligned with local noticing, whereas Write and Induce become increasingly important as the evidence grows less visible and more abstract. This interpretation is consistent with recent syntheses emphasizing the influence of task design, learner characteristics, and type of corpus interaction on DDL outcomes (Huang and Ma, 2025; Pérez-Paredes and Boulton, 2025). The findings therefore connect the cognitive demands reported by learners with the layered account of lexical meaning provided by EUM and support guided concordancing before a gradual transition toward more independent consultation.

### 5.3 Pedagogical implications

The pedagogical value of the present study lies in showing how corpus evidence can be organized and scaffolded for synonym differentiation in EFL classrooms. Because the distinctions between near-synonymous verbs often reside in recurrent patterns of collocation, colligation, and semantic preference rather than dictionary meanings alone, the findings point to several implications for EUM-informed DDL-based synonym instruction.

First, DDL can make vocabulary instruction more evidence-based by exposing learners to authentic examples of language use and encouraging them to reason from data rather than rely exclusively on dictionary definitions. In synonym differentiation, however, learner autonomy needs to be carefully scaffolded. The present findings suggest that learners benefited when corpus evidence was made accessible through pre-selected concordance lines, guided observation, and structured recording tasks. In this sense, DDL supported a more active form of vocabulary learning while still preserving the teacher’s role in mediating complex corpus evidence.

Second, the EUM framework offers a useful analytic lens for helping learners move beyond general meaning explanations toward phraseological evidence. For near-synonymous verbs, the relevant pedagogical question is not only what a word means, but also which words it tends to occur with, which grammatical environments it favors, and which semantic domains it is typically associated with. Organizing vocabulary instruction around collocation, colligation, and semantic preference can therefore help learners understand why two words with similar dictionary meanings are not always interchangeable.

Third, OMWI provides a classroom procedure for translating EUM-informed DDL into observable learning actions. The sequence of observing, marking, writing, and inducing helps learners move from concordance evidence to explicit pattern awareness. Observation exposes learners to recurrent usage, while marking draws attention to salient lexical and grammatical clues. Written recording stabilizes emerging findings, and induction encourages learners to formulate broader synonym distinctions. In this way, OMWI links the authenticity of corpus data with the instructional need for input enhancement and structured exposure, a concern also emphasized in usage-based approaches to DDL materials (Römer, 2024).

Finally, classroom implementation of EUM-informed OMWI may require considerable teacher preparation and sustained pedagogical support. The comparatively low willingness to use DDL in future teaching (S20, *m* = 2.64) contrasts with participants’ generally favorable perceptions of its learning value. This contrast suggests that they may not yet have felt ready to transfer an unfamiliar corpus-based procedure from learner use to teacher use.

Although the concordance materials were prepared by the instructor, working with a substantial body of pre-selected corpus evidence may have led participants to anticipate the considerable selection, organization, and scaffolding required to implement similar activities in their own future teaching. This uncertainty may also be related to their limited experience as first-year students and the difficulty they reported in working with less visible patterns such as colligation and semantic preference. Because the questionnaire did not directly investigate the reasons for the S20 response, however, this interpretation remains tentative. At an introductory stage, DDL may therefore be most productive as a complement to traditional vocabulary instruction, supported by pre-selected concordance lines, reusable materials, simplified tools, and appropriate teacher guidance. Guided material-adaptation and low-stakes microteaching activities may further help learners who later enter teaching develop confidence in implementing DDL themselves.

### 5.4 Limitations and future research

Although the study provides evidence for the pedagogical potential of EUM-informed DDL-based scaffolded concordancing, the findings should be interpreted in light of several methodological and contextual limitations, which also indicate directions for future research.

First, the study involved two relatively small groups of 30 participants, and most participants were female first-year English majors. This sample composition may limit the generalizability of the findings. Future studies should recruit larger and more diverse samples across different institutions, majors, proficiency levels, and gender groups in order to examine whether the effects of DDL-based scaffolded concordancing are stable across learner populations.

Second, the study was conducted in a classroom setting and did not include a delayed posttest. Therefore, the study cannot establish whether learners retained their gains over time, and the findings cannot be generalized directly to online, blended, or self-access learning environments. Future research should include delayed posttests and compare classroom-based, online, and independent corpus consultation contexts. A further limitation concerns measurement. The contextualized synonym test assessed overall synonym differentiation rather than collocation, colligation, and semantic preference as separate outcomes, while the questionnaire captured learners’ perceptions rather than demonstrated performance. With 28 valid responses, the questionnaire was analyzed descriptively, and its reliability and validity remain to be established. Future studies should supplement the contextualized synonym test with receptive and productive measures of individual EUM dimensions and validate the questionnaire in a larger sample.

Third, the study focused on collocation, colligation, and semantic preference as the main EUM-related dimensions of synonym differentiation. Other dimensions, such as semantic prosody, register distribution, frequency, genre, and degree of formality, were not systematically examined. Future studies could extend the EUM-informed instructional framework by incorporating these additional dimensions and testing whether learners can use them to distinguish near-synonymous items more precisely.

Fourth, the target vocabulary was limited to five pairs of near-synonymous verbs. Future research could apply the same instructional framework to adjectives, adverbs, nouns, phraseological units, or larger synonym sets involving more than two lexical items, so as to evaluate the broader applicability of scaffolded concordancing for vocabulary learning.

Finally, selecting suitable concordance lines and developing classroom materials involved substantial teacher preparation, which may limit the scalability of DDL-based instruction. Future research could explore semi-automated ways of selecting pedagogically appropriate concordance examples, compare direct and indirect DDL approaches, and investigate how corpus tools can be adapted to reduce preparation demands while preserving the benefits of guided discovery learning.

## 6 Conclusion

The present study investigated the effects of EUM-informed DDL-based scaffolded concordancing on EFL learners’ differentiation of near-synonymous verbs and explored learners’ perceptions of the OMWI framework in supporting awareness of collocation, colligation, and semantic preference. The quantitative results showed that both traditional vocabulary instruction and the EUM-informed DDL-based approach improved learners’ performance, with the Experimental Group achieving significantly larger learning gains than the Control Group. The questionnaire results further suggested that learners generally perceived the OMWI procedures as helpful for observing concordance evidence and noticing phraseological patterns, although some aspects remained challenging. Theoretically, the study contributes to corpus-informed vocabulary learning by showing how the Extended Unit of Meaning can be operationalized in classroom-based synonym instruction and how Mark and Write can mediate the progression from observation to induction. Pedagogically, it suggests that prepared concordance lines, coloured annotation, structured recording, and guided induction may help novice corpus users attend to phraseological patterns associated with near-synonymous words.

## Data Availability

The raw data supporting the conclusions of this article will be made available by the authors, without undue reservation.
